# Using traditional healers to treat child malnutrition: a qualitative study of health-seeking behaviour in eastern Ethiopia

**DOI:** 10.1186/s12889-022-13323-5

**Published:** 2022-05-02

**Authors:** Ketema Degefa, Adugna Tadesse, Caroline Ackley, Lola Madrid, Nega Assefa, Markus Breines, Kasthuri Sivalogan, Maria Maixenchs, John Blevins

**Affiliations:** 1 College of Health and Medical Sciences, Haramaya University, Harar, Ethiopia; 2 Brighton and Sussex Medical School, Brighton, UK; 3 London School of Hygiene & Tropical Medicine, London, UK; 4 Emory Global Health Institute, Emory University, Atlanta, GA USA; 5 ISGlobal, Hospital Clínic-Universitat de Barcelona, Barcelona, Spain

**Keywords:** Malnutrition, Health-seeking behaviour, Ethiopia, Child mortality, Healers, Healthcare system, Qualitative

## Abstract

**Background:**

Malnutrition among children under five years of age is a major public health issue in many low and middle-income constrained countries. According to WHO, 5.3 million under-five children die every year and about 45% of these deaths are linked to malnutrition. While it is clear that poverty and lack of food are important factors in children’s malnutrition, less is known about the ways in which local conceptions of malnutrition affect parents’ treatment choices. In Ethiopia, child malnutrition is a severe public health problem and a common cause of child death, and this paper explores the local views of malnutrition and how these shape people’s health-seeking behaviour.

**Methods:**

The study was conducted in eastern Ethiopia from December 2017 to January 2019, conducting interviews and focus group discussions to explore different views and treatment options malnutrition. The study used grounded theory because it allows new and unexpected themes to arise from the data. Researchers’ assumptions on local terminologies of child malnutrition are also controlled as a principle of ground theory.

**Results:**

Child malnutrition was not only perceived to be related to lack of food but was understood in a wider local conceptualization of health and illness. Parents often relied on healers because they are long-standing members of the community, possess indigenous knowledge, and cost less than other options. Because health professionals and the community perceive and speak of health very differently, people often do not seek support from health services. The misalignments between how health professionals and healers diagnose and treat malnourished children have implications on the possibilities to implement change to reduce malnutrition.

**Conclusions:**

Through an exploration of people’s own terminology and understandings of what a malnourished child is, as well as the underlying reasons for their illness, this paper explores how people understand malnutrition symptoms and why many tend to rely on healers rather than seeking care from health centres.

## Background

Poor nutrition is a key driver and risk factor for child disease. Improving child nutritional status demands sustained and effective programming and engagement [1], but eradicating malnutrition has proved difficult [2]. Adequate maternal nutrition before and during pregnancy and lactation; optimal breastfeeding in the first two years of life; nutritious, diverse and safe foods in early childhood against malnutrition are insufficient to anticipate reaching goal 3 of SDGs aimed to ensuring good health and promoting healthy lives by 2030. Among children under five, 149 million are stunted (low height-for-age), over 49 million are wasted (low weight-for-height) and nearly 17 million are severely wasted, a majority of whom live in low-income countries [2]. Estimates indicate that 5.3 million under-5 children die every year and about 45% of these deaths are linked to malnutrition [2]. Despite the scale of the problem, little is known about how people themselves understand malnutrition and how these views and intra household dynamics affect the decisions to seek support for malnutrition. In response, this paper shifts the attention from issue of access to resources to consider health-seeking behaviour related to malnutrition.

Social scientific studies have brought attention to the socio-cultural aspects of malnutrition. It has become clear that in some contexts malnutrition is not seen as a health problem but rather as a normal part of children’s growth [3]. Similarly, Mengesha argue that food is not only about nutritional value, but that people’s choices about what, when, where, how and with whom to eat are influenced by religion, gender, status and other factors [4]. In addition to food practices, parent’s conceptions of malnutrition of children are cultural [5]. These studies suggest that malnutrition is embedded in social, cultural, economic, and political relations, and as such, the nutritional status of members of a household is not only determined by nutritional content but also cultural patterns that govern the diet system and values within the community at large. This differs from biomedical perspectives where malnutrition is seen as an outcome of poor diet or severe and repeated infections, and people’s understandings may not align with those of biomedicine as they have their own understandings of causes, symptoms and identification of malnutrition [6]. However, little is known about how these dimensions of malnutrition affect people’s health-seeking behavior.

Malnutrition of children has been a serious problem in Ethiopia for many years [7]. Socio-cultural practices, intra-household food distribution, seasonal food insecurity are important causes of child malnutrition [8], but in Ethiopia malnourished children who are stunted/wasted are sometimes seen as normally growing children [9]. Poor health-seeking behaviour negatively contribute to under-five child death and appropriate care-seeking practices have the potential to substantially reduce child mortality related to malnutrition. According to Ali’s study of health-seeking behavior of the Afar pastoral community, the choice of health care institutions for treatment are influenced by beliefs about the causes of diseases, trust on the cure of medication, the influence of others, attitudes towards care services, and socio-cultural factors [6]. While people sometimes consider malnourished children to be growing as normal, their health-seeking behaviour related to children’s malnutrition have received little attention. However, it is clear that their views of malnutrition affect their actions and it is necessary to explore these to develop strategies to alleviate the issue.

This paper emerges from the Child Health and Mortality Prevention Surveillance (CHAMPS) program, which is a seven-country network established by the Bill & Melinda Gates Foundation to identify the causes of death in children in communities with high rates of under-5 mortalities [10]. The program carries out both mortality and pregnancy surveillance, and mortality surveillance employs minimally invasive tissue sampling (MITS) to gather small samples of body fluids and tissue from the bodies of children who have died [11]. Moreover, the clinical and verbal autopsy data collected from the Child Health and Mortality Prevention Surveillance sites indicated malnutrition as one of the main causes of under-five child death [12]. As part of the mortality surveillance in Ethiopia (one of the CHAMPS sites), a qualitative study explored the local views of malnutrition and related health-seeking behaviour, which identified that several deaths from malnutrition could be attributed to delays in seeking professional health care or not seeking care at all. In order to understand local views of malnutrition, this paper explores how people view malnutrition, how families seek healthcare for malnourished children and how traditional healers treat malnutrition.

While child malnutrition is a major problem in many countries, local views and health-seeking behaviour of child malnutrition remain insufficiently considered. In addition, people often do not seek support from health services and rather rely on healers, which has implications for possibilities to treat malnourished children. By doing so, it shifts the attention from nutrition to the socio-cultural dynamics that lead people to ignore malnutrition among their children or to rely on healers rather than on the formal health system.

## Methods

### Setting

This study was conducted in the Kersa Health and Demographic Surveillance System (HDSS) site in the Oromia region of eastern Ethiopia. The Kersa district has 38 Kebeles (the smallest administrative unit in Ethiopia), of which 24 were included in the HDSS (three are urban and 21 are rural). The total population of the district is estimated to be 172,626 of whom 6.9% are urban dwellers people [12].

93.8% of the study population practice agriculture as their primary means of livelihood and the remaining 6.2% are urban dwellers. The cereal crops most commonly grown are sorghum, maize, wheat, barley and pulses in their order of importance. Khat and vegetables are the main cash crops. Cattle, goat and sheep are common in the community. The main economic activities are food crop production, cash crop (Khat) production and livestock production.

### Design

The Child Health and Mortality Prevention Surveillance (CHAMPS) network, with seven sites in Africa and South Asia, aims to identify and track definitive causes of under-five child mortality in regions where it is highest, and to generate and share high-quality data to inform policy and public health action. CHAMPS began work in Ethiopia in August 2017, conducting formative research and community engagement activities. While assessing community perceptions on maternal and child health problems, malnutrition was identified as a top priority. As a result of this study new local views of malnutrition was explained by participants.

The CHAMPS Social-Behavioral Science conceptual framework is based on ethnographic method. Ethnography is an approach seeking to understand how a group of people give a meaning for particular topics. which result in an understanding of certain cultural behaviour and practices [13] and this approach allows opportunities to explore people’s day to day lives and understand local meanings of malnutrition. Ethnographic principles were used to develop interview guidelines and data collection procedures. First, the researchers established relationships with study participants and then developed interview guides based on the research questions. Second, face to face interview was used, then probing was also used to further understand vague answers and to know more details.

Finally, the study used an iterative approach to make the analysis ongoing based on fieldwork observation and emerging themes, resembling a spiral process rather than a circular or linear process [14]. Thus, the topic areas in the guideline included local views related to malnutrition treatment, conditions in which traditional healers are consulted, and categories of malnutrition and health seeking towards them.

### Participants and data collection

Before actual data collection, the team in CHAMPS Ethiopia received training on qualitative research, data collection tools, participant selection, interaction with participants and how to be reflective, and minimize subjectivity [15]. This in turn supported to control the quality of the study. The study was part of broader objectives of CHAMPS program and the main aim is to understand local views of childhood malnutrition. From the preliminary findings of the mortality surveillance, malnutrition is one of the major causes of child death and local views are not addressed well. The study used this initial assumption to conduct the study.

In order to choose the study participants the team followed Participatory Inquiry Into Community Knowledge of Child Health and Mortality Prevention (PICK-CHAMP), a framework to select participants from diverse groups who were representative of community groups, activities, and/or individuals [16]. The study used purposive theoretical sampling techniques and participants were selected based on their prior knowledge of the research questions and the majority of the mothers who were interviewed received care either from traditional healers or healthcare.

For this study, in order to select the participants for interviews and Focus Group Discussions, researchers worked closely with local Health Demographic Surveillance data collectors who live in the community and they helped facilitate the recruitment process. Data collectors recruited participants who had experience in local treatment of child malnutrition and mothers admitted at children nutrition rehabilitation center. Moreover, to triangulate the data people who could affect or influence community members’ perceptions and practices around child health also participated in the study. The researcher met the study participant in person, asked for written consent and conducted data collection. Moreover, the study data collection has been conducted by the authors at participants homes and health facilities.

The one-to-one interviews were conducted in a private locations preferred by the respondents, such as their homes. Key informant interviews (total *n* = 12) were conducted with religious leaders (*n* = 2), a traditional birth attendant (*n* = 1) and health extension workers (Health extension workers are people who have received basic training to support the primary health care unit at the health post level or grassroots level) (*n* = 2). Children and women affairs experts (*n* = 2), and mothers of children admitted to the nutrition rehabilitation unit at Kersa and Water health centre (*n* = 5) were participated in the study. The interview participants were limited to 12 people due availability of the participants who had experience with child malnutrition healing practice and the sensitivity of the topic.

Four Focus Group Discussions (FGD) with 8–10 participants were held with pregnant women (*n* = 1), religious leaders (*n* = 1), and mothers of children under five years old (*n* = 2). FGDs conducted in Health Demographic Surveillance offices. With regard to time, the interviews took 35–40 min on average, while the FGDs lasted from 45 to 65 min.

In addition, the study conducted participant observation in multiple settings at community over a two year period level to understand the local views of malnutrition and the healing process. The observation target included; mothers who providing care for malnourished children at rehabilitation center, nutrition screening campaigns at community level and families home who have under five children. Therefore, the participant observation was done to see what people say and actually do. In addition this, data was used to triangulate interviews and FGDs data.

During the data collection process, the researchers who collected data spoke the local language and this contributed to clearly understanding the conversation with participants and probing for further questions. In addition, this supported the data analysis process. However, knowing the local language does not guarantee against bias. It is clear that most of the time mothers are the ones who look after their children and they are often too busy to give interviews and shy to talk about sensitive information like childhood malnutrition. Moreover, gender differences between the researcher and the participants also create obstacles for knowing why traditional healers are preferred to modern medicine. In order to reduce this bias at the time of data collection and analysis, multiple people participated to address reliability, reduce bias and quality control.

### Data analysis and interpretation

In this study grounded theory is used to understand what are local views of child malnutrition in children under five years and how people view under-five malnutrition and their health-seeking behaviour. Grounded theory, which enables researchers to develop theory from data [17], guided the research and the analysis of the data. It is a holistic and flexible approach to construct theory through analyzing qualitative data. It starts using inductive data, relies on comparative analysis, involves simultaneous data collection and analysis, and includes strategies for refining the emerging analytic themes [18].

Therefore, the value of grounded theory is to explore basic social processes and to understand the multiplicity of interactions that produces variation in research findings. In this regard, the constructive grounded theory is applied as an inductive approach with an ongoing process of simultaneous data collection and data analysis.

The study included constructivist grounded theory strategies. It allows to explore multiple standpoints related to childhood malnutrition treatment. The study used several strategies as part of grounded theory; collecting data in iterative stages, using theoretical sampling, coding the data, data interpretations to link with the theoretical foundation of the study, constructivist ground theory [19]. Furthermore, the study used grounded theory in order to see the difference and communalities of themes in data analysis process, and allows new and unexpected themes to arise from the data. In addition, it helps reduce researchers’ assumptions. After data was collected, themes were generated based on the views of the participants towards child malnutrition including local concepts of malnutrition and health seeking behaviour.

Data was organized, edited, and analyzed using the NVivo, version 11 software package. To address reliability and reduce bias in analytical interpretation, different researchers were involved in data interpretation and quality control. An iterative process has been used to sort and analyze interviews, focus group discussions and observations data. Moreover, the observation report was also uploaded in the NVivo project and coded and go through an iterative approach and analysed continuously until the end of data collection.

### Ethical considerations

This protocol was approved by the institutional ethics committee of the College of Health and Medical Sciences, Haramaya University and by the National Committee at the Ministry of Sciences and Higher Education (30.10/70/2018). Written informed consent was obtained in the local language from the participants by signing (or thumb-printing in case they were illiterate) on the consent form. The study participants were informed that there was no risk of participating in this study, that there would not be any direct payment for participating and that the information they share would be protected. All participant names are pseudonyms.

## Results

### Socio-demographic characteristics of the study participants

A total of 172 participants took part in the study. Sixty-three per cent (63.3%) were female and more than half were above 40 years old. Seventy-seven per cent (77.3%) of them never attended formal education and the majority 96.5% of the participants were Muslim (96.5%) and married (87.7%) (Table 1).

**Table 1:** Socio –demographic characteristics of study participants from December 2017- to January 2019, Kersa District, Eastern Ethiopia

Socio-demographic characteristics	Participants (n)	%
Sex		
Female	109	63.3%
Male	63	36.6%
Total	172	100%
**Age group**		
> 40	89	51.7%
31–40	46	26.7%
21–30	29	16.8%
≤ 20	8	4.6%
Total	172	100%
**Educational status**		
No formal education	133	77.3%
Grade 1-8^th^	17	9.8%
Grade 9-12^th^	13	7.5%
More than Grade 12^th^	9	5.2%
Total	172	100%
**Religion**		
Islam	166	96.5%
Orthodox Christianity	5	2.9%
Total	172	100%
**Marital status**		
Married	151	87.7%
Widowed	21	12.2%
Unmarried	9	5.2%
Total	172	100%

### Local concepts of malnutrition

The research participants had a range of local terms to identify signs of malnutrition and to describe the symptoms. According to participants common signs of malnutrition signs include swollen belly and legs, discolored hair and skin, thinness, discolored eyes, scabies, yellowish eye color, weakened bones and tooth decay, among other symptoms. The causes for such symptoms were explained by parents as well as healers through a range of different local concepts that were associated with malnutrition. In addition, the study used the ground theory approach as a result new research themes emerged which includes local views of malnutrition, health seeking behaviour towards malnutrition.

Almost all respondents mentioned the local term ‘fadhido’ to describe malnutrition and considered as sign of poverty. This term was commonly used to describe generic name for the malnourished child. The research participants described different symptoms and signs of fadhido, such as swollen body and belly, watery diarrhea, discolored hair and skin, significant thinness, poor appetite, bigger head, weak bones, need for heat, and eating of soil. Symptoms could also include fever, irritability, vomiting, coughing, discolored eyes, scabies, weak immunity, and tooth decay. In addition, Kemiya, who was about 45 years old and had one child, explained: “Children who have symptoms of fadhido are unable to suck breast”. Usually the signs and symptoms of fadhido are easily identifiable through physical symptoms, but it was not seen as a serious disease that needs medical interference unless a malnourished child's health situation deteriorated further. Part of the reason for this is that fadhido was associated with poverty and was seen as a failure to feed children. An approximately 65-year-old woman Fetiya who was a traditional birth attendant and had experience of treating child malnutrition explained; “Children becomes fadhido because of food shortage in the families”. Because of the association with poverty, families would feel ashamed if their children showed symptoms of fadhido and hesitate to seek support from others.

Hudufor is another local term for malnutrition and usually characterized by a swollen belly and legs. A female healer, Alifeya, who had many years of experience in malnutrition treatment explained that “Causes of hudufor are said to be abdominal parasite reproduction and considered to be the source for stomach pain”. This lowers the child’s interest in food, but some of the participants explained that children eat soil. 25-year-old Amdiya who had a 9-month old baby who had been delivered with the help of traditional birth attendants explained that “I take the child to the healer for hudufor practice to protect him from malnutrition”. Amdiya had previously lost a child who had moderate malnutrition, but rather than relying on health centers she continued to use healers to prevent hudufor.

Another concept respondent used in relation to malnutrition was Waan shimbirro. A mother around 40 years old called Juwareya and living in the study site explained that waan shimbirro means “if a bird flies over a pregnant woman the baby she will give will be malnourished or die”. The passing bird is believed to cause a loss of appetite in the baby, as well as swelling of the body, yellowing of the eyes, and a persistent cry. These findings show that the cause of malnutrition is not only merely related to food shortage or poverty but traditional views where other factors affect children’s health. In the case of waan shimbirro the cause was not attributed to poverty, but rather to factors beyond people’s control.

There are also other concepts used to describe different symptoms associated with malnutrition. Some were classified based on the symptoms, such as Qaamni gadi adeemu (stunting) was also explained as a baby failing to grow appropriately as it passed through developmental stages. The condition is characterized by shorter height and extreme thinness. The effects of the condition include a prolonged time period for a baby to begin crawling, standing, or walking due to what is referred to as ‘weak bones’. Another, Ciiniinaa garaa (stomach distension), was considered to arise from shortcomings in mothers’ care for their children.

Like with other views of malnutrition, many of these conditions were seen as normal challenges of children’s developmental stages. Some respondents explained that families resist responding to these symptoms and only take action or visit traditional health practitioners when the symptoms persist for a long period of time or the baby become too weak to walk and eat. Several research participants said that mothers only take children who are extremely thin or edematous to health centers. On the other hand, Abdela, an approximately 35 years old man, explained that “Malnutrition is shameful because the community considered you as the poor”. The participants reveled that there is a feeling of shame for the families with malnourished children. It considered shameful because the disease is either a sign of poverty or negligence of the families, which suggests that some people in the study area preferred not referring to their children’s malnutrition as a disease to not to be labeled as poor.

These different concepts and local views illustrate that malnutrition is not only related to lack of food, but are rather embedded in a range of cultural processes. However, also the shame of being poor and local ideas of other factors influencing children’s health, such as the impact of a bird flying over a pregnant woman. These illustrate that malnutrition is not merely a biomedical phenomenon but has very specific meanings in this context, as indicated by the different malnutrition terminologies above. These local perceptions affect how people respond when children show symptoms of malnutrition, which we turn to next.

## Health-seeking behaviour for malnutrition

Several factors affected the health-seeking behaviour related to under-five child malnutrition. Respondents frequently identified home treatment as the starting point when a child became unwell. It was often the mother who first identified that a child was ill and she would then consult others in the household to decide on a course of action. At the early stages, families treated their children themselves. However, if the condition did not improve they would visit a traditional health practitioner who would provide medication in the form of herbs and massages. A female health extension worker called Zahara who usually worked with mothers in primary care and taught on prevention of malnutrition explained that “malnutrition is not considered to be a disease in its early stages and a malnourished baby would only be taken to health facilities when the child’s condition reaches a critical point”. To reach such a point the child would have to be unable to walk or eat. However, also the local perceptions of malnutrition as ‘normal’ affected families’ decision to take a malnourished baby to health facilities. Also the opinions of neighbors or relatives influenced decisions about whether to treat malnutrition at home, obtain medication, or take a sick child to healers. Sometimes families also consult elders and religious leaders for advice on what kind of treatment to use. Health-seeking behaviour related to malnutrition did not take place on an individual level, but was rather entangled in a range of social relations.

Many respondents stated that economic factors as well as the lack of access to health centers made them rely on healers instead of health centers. The families that faced child malnutrition often faced significant economic challenges. Mothers were commonly responsible for household chores, working in the fields (especially around planting or harvest times), and looking after their children. Taking care of sick children had to be balanced with the other responsibilities because of pressing economic demands. Usually many mothers did not have sufficient time to travel to health centers to get treatment for their sick children as that would require a lot of time as well as expenses for transport (if available at all). In order to find solutions to the problem, local people recommended that health facilities teach communities to choose healthy behaviors. Local health extension workers suggested raising understanding and awareness of malnutrition as a disease while empowering mothers to seek treatment. In addition, they advised that further interventions should encourage behavior change to ensure that families seek health care for malnutrition.

People also drew on support from relatives, neighbors and wider community to provide health care for their children. Often grandmothers help out to look after young children when mothers give birth or are ill, but also during cases of malnutrition. One grandmother who had brought a malnourished child to a health center explained: “I’m the grandmother of this child, she is unable to eat and drink. When we try to feed her, she vomits soon. Her mother gave birth two weeks ago and is unable to visit a health facility to treat the baby. She delegated me to do that. This baby is a nine-months old”. In this case, the grandmother went to the health center because the child was seriously ill and healers had not been able to treat her. However, in many cases the use of health centers was limited, but because of the healer’s treatment had not worked and the baby’s condition was deteriorating this was used as a last resort.

While there are health posts in every sub-district, they have very limited medical supplies and many research participants questioned their skills. More advanced health-care facilities, such as health centers, are clustered in three or four sub-districts, but are difficult to access for people who live far away. Another reason why families hesitate to visit health facilities is that the family feels shameful that their child is malnourished because it is associated with poverty and believed to mainly occur during famines. The poor facilities and limited opportunities for accommodation meant that some of the families that went there ended up leaving the centers before the treatment was completed. These factors then contributed to people either ignoring the symptoms up to a certain point or to rely on healers.

Traditional healers who recognized by local people as a knowledgeable to treat malnutrition hold a strong position and these are more commonly used than health centers. Ferida, a woman around 40 years, had seen many cases of malnutrition and explained “When the child’s body has swollen belly and legs, we say the baby had abdominal parasite reproduction in his stomach and the parasites reproduced in the abdomen which results in a swollen belly, and then gradually causes stomach pain. After some days, we go to healers to relieve the child from the problems.” This was a common practice for treating hudufor and the treatments healers would offer differed significantly from biomedical practice. In the case of swollen belly and legs, the healers (often elder women) would often insert short bits of wood from a root and rotate them inside the anus so that bleeding occurs for the purpose of making the parasites bleed out. However, the clinical health practitioners said the traditional treatment of rotating the root into the anus may create infection and that infection developed into hemorrhoids: ‘Hudufor treatment is the main causes for hemorrhoids’’. Another treatment for malnutrition called Chuma re’e is where the stomach of a slaughtered goat is placed on the abdomen of the child. These practices were common and parents accepted them as normal ways of improving children’s health and well-being.

The government prohibits healers from treating malnutrition, but such practices are still common. One traditional birth attendant, Fatuma, explained that people use healers secretly because they trust them more than the biomedical care in health centers. Because of these restrictions, many of the research participants initially hesitated to speak about their use of healers, but as discussions progressed many revealed that they used healers because they trusted them more than health center workers and they suggested that there are plenty of traditional health practitioners capable of treating the different kinds of malnutrition. For example, when a child gets swelling of the body and its eyes become yellowish, healers’ treatment consists of burning the child’s chest, bottom of the belly and the bottom of the back. After burning in these places, the child will be fed goat milk mixed with a powder made from the dried once, finely crushed root of a plant called Baal tokke (local plant) and then the child is expected to recover from waan ijoolle (one kind of malnutrition). However, these practices sometimes made it difficult to bring their children to health centers if symptoms worsened because, as one health practitioner called Jemal explained, parents sometimes hesitated bringing their children to the health centers they feared that they would be reported to the child and women affairs office if the marks of the burning on the child’s body could be seen. As such, the illegality of traditional health practices could prevent people from seeking help from health centers.

Evidently, biomedical health professionals and healers treat malnutrition in very different ways. While there were research participants who thought the health centers was the most reliable place to obtain treatment, others found that healers produced better results. Healers were trusted because they are long-standing members of the community, possess indigenous knowledge and cost less. In addition, the long distance to health centers and challenges related to cost and transport made healers the most feasible health care option for treating malnutrition, illustrating that the use of healers who perceive the symptoms in similar ways as parents is a more feasible strategy for them.

## Discussion

In sub-Saharan Africa and South Asia, there are many governmental programs and non-governmental organizations working on improving nutritional status of children. However, the number children dying because of malnutrition is high and this paper reports how people in a rural area of eastern Ethiopia perceive and treat malnutrition. In other contexts, it has been pointed out that there are a wide variety of local conceptions of malnutrition. For example, Muraya found that kwashiorkor was the term mostly used to indicate all symptoms related to child malnutrition in Kenya [3]. Likewise, mamarcha is a generic name of illness related to childhood malnutrition in India even though malnutrition in itself was not considered to be a serious health condition in children [20]. Like in Ethiopia, such concepts have implications for understanding how parents perceive and treat malnutrition. However, less attention has been given to cultural practices in policy formulation regarding children’s health and malnutrition. In this paper, it has been shown that local views and practices are central for how people perceive and respond to malnutrition.

While the participants in this research were well informed about how feeding habits, maternal care and poverty could lead to malnutrition as well as the various symptoms of malnutrition, there were several reasons why they relied on healers instead of health centers. This included, for example, the trust in healers over biomedical treatment and preference for medicines that they were familiar with. Study participants informed healers are using plant roots from home gardens and nearby forest patches for hudufor treatment. Similarly, in southern in Ethiopia, traditional medicinal plants are used for child malnutrition treatments [21]. These findings are similar to those from India where they found that people relied on traditional and cultural practices for health care and nutrition for newborns, infants, and young children [22]. Moreover, the association of malnutrition with poverty made people feel ashamed and hesitate to seek support when their children, and some of the healers’ practices (such as burning specific spots on the child’s skin) could create problems for the family if seen by professional health workers. Other factors such as long distances to health centers and financial challenges also impacted their treatment choices.

These findings resemble findings from other parts of Ethiopia and provide insights into the importance of adapting health interventions to the local contexts. A study from southern Ethiopia suggested that a significant proportion of caregivers did not seek care for childhood illness and most caregivers did not know where and when to seek care [23]. A study conducted among the Afar pastoral community found that people often preferred self-medication, but also that their treatment choices were influenced by their views about the causes of diseases, trust that medications would provide a cure, availability of required medicines, the cost of health care services, the influence of family and relatives and attitudes toward health care services [6]. Combined with the findings in this paper, this suggests that top-down approaches to reducing malnutrition are unlikely to succeed. Instead, the findings highlight the importance of international health policymakers and health organizations paying more attention to local views and practices to develop appropriate interventions that are based on community engagement and involvement.

The research participants themselves also suggested intervention activities that could encourage healthy feeding practices and reduce malnutrition. These included empowering women in gardens activities and improve livelihoods, nutrition education sessions through food demonstration events both at the community and health facility levels.

## Limitations

The study was conducted in rural areas in eastern Ethiopia and while the results are of relevance for malnutrition there, the findings are not necessarily applicable to other contexts. In addition, studying malnutrition is sensitive among traditional healers and the researchers faced challenges to recruit those who treat malnutrition in the community. Local law prohibits traditional healers to treat malnutrition and many of them were concerned about the researchers reporting their practices to law enforcement. The challenges of recruiting traditional healers was an important limitation of the study and we have limited data on their views on treatments of malnutrition.

## Conclusion

Incorporating local views and practices in nutritional program activities is essential. To improve children’s nutritional condition, health practitioners need to work closely with local community members, such as religious leaders, elders, healers, traditional birth attendants, and other people who have knowledge and respect for local views. Therefore, well organized behavioral change practices should not be imposed, but rather be developed in collaboration with local communities. While nutrition messaging and health education is crucial to improve knowledge, attitude, and practices to malnutrition, understanding local views and health-seeking behaviour will facilitate better collaboration and co-construction to an endemic challenge in many countries.

## Data Availability

Due to the sensitive nature of the data collected for this study, including but not limited to; detailed and in-depth information on the families under study, information on family tensions and dynamics, and the status of individual children’s health, it is not possible to deposit the entire data set in a publicly available repository. Data for this work will be held by the Haramaya University Hararge Health Research Partnership under managed access. Data are however available from the corresponding author (degefaketema30@gmail.com) upon reasonable request.

## Abbreviations

WHO: World Health Organization
SDGs: Sustainable development goals
CHAMPS: Child Health and Mortality Prevention Surveillance
MITS: Minimally invasive tissue sampling
HDSS: Health Demographic Surveillance Site
TBAs: Traditional birth attendants
